# Intestinal Organoids as a Novel Complementary Model to Dissect Inflammatory Bowel Disease

**DOI:** 10.1155/2019/8010645

**Published:** 2019-03-19

**Authors:** L. Schulte, M. Hohwieler, M. Müller, J. Klaus

**Affiliations:** Department of Internal Medicine 1, University Medical Centre Ulm, Ulm, Germany

## Abstract

Inflammatory bowel diseases (IBDs) include colitis ulcerosa and Crohn's disease, besides the rare microscopic colitis. Both diseases show a long-lasting, relapsing-remitting, or even chronic active course with tremendous impact on quality of life. IBDs frequently cause disability, surgical interventions, and high costs; as in other autoimmune diseases, their prevalent occurrence at an early phase of life raises the burden on health care systems. Unfortunately, our understanding of the pathogenesis is still incomplete and treatment therefore largely focuses on suppressing the resulting excessive inflammation. One obstacle for deciphering the causative processes is the scarcity of models that parallel the development of the disease, since intestinal inflammation is mostly induced artificially; moreover, the intestinal epithelium, which strongly contributes to IBD pathogenesis, is difficult to assess. Recently, the development of intestinal epithelial organoids has overcome many of those problems. Here, we give an overview on the current understanding of the pathogenesis of IBDs with reference to the limitations of previous well-established experimental models. We highlight the advantages and detriments of recent organoid-based experimental setups within the IBD field and suggest possible future applications.

## 1. Multifactorial Pathogenesis of Inflammatory Bowel Diseases

Inflammatory bowel diseases (IBDs), mainly summarizing Crohn's disease (CD) and ulcerative colitis (UC), are characterized by chronic relapsing-remitting or continuously active inflammation of the bowel, sometimes accompanied by extraintestinal affections, including diseases of the liver, skin, joints, or eyes.

UC affects the colon with an exclusively mucosal inflammation that almost invariably involves the rectum and spreads continuously to the variable segments of the colon, causing ulcers and leading to bloody diarrhea, accompanied by abdominal pain and signs of systemic inflammation like fever. In severe UC, a septic disease and a colonic distension called toxic megacolon with imminent perforation can develop, with possibly fatal consequences. Long-term complication is first of all the increase in risk for colonic carcinoma [1] especially in patients with an affection proximal to the colon sigmoideum. Furthermore, primary sclerosing cholangitis is associated in about 10% of cases, causing cirrhosis of the liver and its complications.

CD, on the other hand, can affect any part of the gastrointestinal tract from oral cavity to perianal skin, with the distal ileum being the most commonly affected part of it. The disease afflicts the patient with pain and diarrhea. The inflammation in CD is transmural, giving rise to fistulas, abscesses, and strictures, which often lead to the need of surgical resection; affection of the small intestine also leads to malassimilation and malabsorption.

The highest prevalence of IBD is found in highly developed countries, where about 300/100,000 people are affected by each UC and CD [2], with a markedly increased risk for relatives of affected persons [3]. Given the young age of onset of 15-35 years, the associated disease-related reduction of the quality of life [4] and the high morbidity [5, 6], the impact on these young patients is massive. Adding the high direct and indirect costs of the IBD [7] makes them one of the five most expensive gastrointestinal diseases [7, 8]. The need for research on them is obvious.

The current pathogenetic model of IBD is based on an inappropriate response of the hosts' immune system to intestinal microbial factors, in part as the consequence of an ineffective barrier between luminal flora and subepithelial tissues and in part because of an imbalance in the immune reaction of the mucosal immune system [9, 10]. Antibiotics have been used to treat acute IBD flares for a long time, rising suspicion about a crucial role of bacteria in its pathogenesis. Already 20 years ago, it was shown that most mouse models for IBD did not develop intestinal inflammation in germ-free conditions [11–13], but even with current highly detailed techniques of microbiome analyses, no specific pathogenetic organism could be identified [14] and in only one single mouse model a transmittable “colitogenic” flora could be shown; nonetheless, there are specific changes in the composition in the intestinal flora of IBD patients. Further, supporting the role of bacteria in pathogenesis, it was found that in IBD, in contrast to healthy controls, bacteria were able to penetrate the mucus layer [15], maybe in part because of a differing composition of the mucus [16]. Next, bacteria must trigger an immune reaction to cause the intestinal inflammation. Although studies showed increased mucosal permeability in IBD [17], associations with genes involved in mucosal integrity [18–20], an oligoclonal T cell population in the lamina propria [21] suggestive of an antigen-driven immune reaction, and more recently a disruption of the subepithelial band of lamina propria macrophages [22], mucosal adherence or penetration by bacteria could not be shown [15]. Instead, epithelial cells themselves are able to process and present antigens [23], and the inflammatory response itself is able to disrupt the epithelial integrity [24, 25]. Despite these intriguing findings, it seems that they are only part of the story, since none of those risk factors alone is sufficient to cause IBD. Instead, it is likely that imbalances in the host immune response lead to the morbid hyperinflammation in IBD.

A strong hint to a dysregulated immune response was the discovery of IBD1 mutations on chromosome 16 in CD. NOD2, product of IBD1, is an intracellular protein that activates NF*κ*B in response to bacterial fragments. A leucine-rich repeat domain with regulatory and sensory functions was shown to be affected by missense and frameshift mutations in a subset of CD patients and was associated with early onset, predominantly ileal and fibrostenotic disease. Furthermore, these mutations could also predict the phenotype of CD [26–30]. On a functional level, two different mechanisms could be learned from mouse models. NOD2 deficiency led to MDP resistance of macrophages and a reduced production of defensins by Paneth cells, suggesting a reduced mucosal defense against luminal bacteria. On the other hand, mice with the human-like frameshift mutation mentioned above were highly susceptible to DSS-induced colitis with marked increase in intestinal inflammation and NF*κ*B activation, suggesting a dysregulation of the immune response after injury of the mucosal barrier. The normal intestinal macrophages, instead, are typically of a noninflammatory phenotype focused on the phagocytosis and clearance of bacteria [31]. At the same time, none of these models above spontaneously developed intestinal inflammation [32–34]. NOD2 alterations were the first example of alterations in the innate immediate immune response with subsequent discovery of many more. NOD2 belongs to the family of pattern recognition receptors (PRRs), highly conserved receptors for pathogen- and danger-associated molecular patterns (PAMP/DAMP). The PRRs that include the families of RLRs, TLRs, CLRs, NLRs, and others are activated by those PAMPs and DAMPs, triggering an immediate innate immune response. Therefore, they are one of the front links between a pathogen and the host response, some of them have been linked to IBD [35], and are a target of new drugs that are tested in clinical trials (NCT03178669).

One of the key mechanisms of symbiosis between host and intestinal microbiome is immune tolerance, and this central concept of living together seems to be disrupted in IBD. Rare cases of inherited monogenetic disorders lead to IBD-like diseases in humans, for example, mutations in FOXP3, IL10, IL10R, or XIAP. For all of those, a mechanistic link to immune tolerance exists [36]. As outlined above, normal lamina propria macrophages effectively eliminate intruding bacteria without calling for much inflammation; they begin to secrete anti-inflammatory mediators upon bacterial activation [37]. Contrarily, in IBD, myeloid cells of the lamina propria react with a solid proinflammatory response upon stimulation with microbial substances [10, 38, 39]. The switch between tolerance and inflammation is flipped in part by the dendritic cells of the lamina propria in a WNT-dependent fashion [40]. Taken together, the myeloid cells as the first line of defense overreact in IBD. Bridging to the adaptive response, there are also changes in the more recently discovered innate lymphoid cells of the gut. Expansion of ILC1 and NCR-ILC3 cells leads to loss of protective IL-22 and augmented inflammatory IL-17 production [41–45]. The adaptive lymphoid reaction, mainly responsible for the chronic inflammatory reaction, is altered in a harmful way, too. Normal gut-associated lymphoid tissue does not react to commensal bacteria with an intense inflammatory response, but instead actively clears T cells specific to commensal bacteria, a process that is damaged in IBD [46]. There is a growing number of types of lymphocytes discovered as being involved in the inflammation in IBD, accompanied by even more cytokines that are secreted [47]. Th1 mainly react to intracellular pathogens, activating cellular immune response, and are induced by IL-12 and IL-18; both are highly expressed in CD by activated macrophages. The Th1 cells, in turn, then produce IFN*γ*, also detected in high levels in the inflamed CD mucosa; IFN*γ* is considered one of the driver cytokines in CD [48–51]. Th2 cells are drivers of humoral immunity and mount reactions to parasites and are involved in allergic reactions; the main cytokines produced are IL-4, IL-5, and IL-13. Because of high levels of IL-5 and IL-13 in UC, this IBD was assumed to be mainly Th2-driven. However, doubts emerged from the absence of increased IL-4 production and missing evidence for a pathogenetic role of IL-13. Moreover, it was shown that IL-13 is more likely produced by NKT cells in UC [52–55]. Furthermore, while it was assumed in earlier studies that IL-13 drives fibrosis and is the key cytokine in UC, conflicting evidence for this could be found in studies in IBD patients [56–61]. With respect to the oxazolone- and TNBS-induced colitis mouse models, Th1 and Th2 reactions were seen differentially involved; the Th1 response was considered to be more pronounced in TNBS colitis, which resembles some features of CD, whereas the Th2-dependent inflammation was seen in oxazolone colitis, sharing some features with UC [51, 53–55, 58]. Regrettably, targeting neither of IFN*γ* nor of IL-13 and thereby selectively targeting Th1 or Th2 response, respectively, were convincingly effective in treating UC or CD. Therefore, it seems that both pathways can stand in for and control each other [53, 58, 62]. Important progress in the decryption of IBD pathogenesis was made with the discovery of Th17 cells, a type of Th cells producing IL-17A that has been accused to be potently proinflammatory and furthermore being involved in fibrogenesis and MMP production [48, 63–65]. Among further lymphocytes involved in IBD pathogenesis, like Th9, NKT, MAIT, or ILC cells, being far beyond the scope of this review, regulatory T cells oppose the inflammatory reaction and will be discussed only briefly. Tregs mainly produce IL-10 and TGF*β*, which are both able to suppress inflammatory response in effector T cells and induce immune tolerance; in some mouse models, the IBD phenotype could be avoided by Tregs. Although Tregs are infiltrating the inflamed mucosa in IBD, they are obviously unable to stop the inflammation. One possible explanation is the overexpression of the counteracting SMAD7 protein [66–74]. Even adding to the high complexity of this immune response, we learned that the T cells involved seem to be plastic, allowing transdifferentiation of one type to another [75–78]. Furthermore, cells combining the phenotypes of others have been identified, like the Th1/Th17 cell [79]. This plasticity, on the other hand, could open windows for therapeutic interventions.

In genome-wide association studies, more than 200 risk loci for IBD, many of them shared between both CD and UC, have been established [80, 81] and link IBD with additional signaling pathways including autophagy and endoplasmic reticulum stress signaling, but these are again beyond the scope of this review.

## 2. Animal Models of IBD

Much of the knowledge about the immune mechanisms in IBD has been learned from mouse models, but some merits and demerits of these models must be considered. Observing the gut immune response in a whole organism has the advantage that all players of this complex game are in, and therefore, their context-specific action can be explored. However, harsh exogenous or genetic measures must be taken to induce IBD-like disease in mice, which unlikely resemble the factors leading to human disease. Therefore, many mouse models have been developed, and each of them is thought to mirror one piece of the pathogenesis. The most human-like models are congenic models, which are difficult to establish. In some chemical models, extrinsic substances damage the gut epithelium and provoke mainly an acute, innate response of inflammation and repair that can be studied but lack the chronic inflammation that is typically found in IBD; in other cases, gut proteins are modified by haptenating agents and trigger an immune response. Mono- or oligogenetic models shed light onto the specifically targeted pathway but are unlikely to resemble the complex mechanisms in human disease. Finally, adoptive immune transfer models are used mainly to unravel the interaction of lymphocytes [82]. Most of the models develop colitis, and there are only few models with small intestine disease.

### 2.1. Chemical Models: DSS Colitis

First described in 1990 [83, 84], DSS colitis is one of the most frequently used models of IBD. Usually, mice with BALB/c or C57BL/6J background are given 1.5-5% dextran sodium sulfate (DSS) in their drinking water. After some days (typically 6-10 days), the mice develop diarrhea, gross rectal bleeding, and weight loss; the reason for this is an ulcerating acute colitis with infiltration of neutrophil granulocytes. The exact mechanism of action of DSS is not known, but it leads to damage of the epithelial monolayer with increased permeability for luminal bacterial compounds. This acute colitis depends on luminal bacteria, but does not depend on the adaptive immune response, as shown in RAG2-KO or SCID mice [85, 86]. Therefore, this model was most useful in exploring the innate immune response, including the TLR- and inflammasome-dependent pathways [87, 88] as well as macrophage and neutrophil contributions. This has led to some unexpected findings. Since TLRs and MyD88 have well-known inflammatory properties, the increased epithelial permeability in DSS colitis would be expected to lead to TLR activation and therefore to a more severe inflammation. However, mice lacking TLR2, TLR4, or MyD88 developed even more severe inflammation [89], pointing to a regenerative and protective as well as immunomodulating effect of these proteins [90, 91]. The mechanisms of regeneration, then, can also be studied in the DSS colitis model and have led to the discovery of the involvement of GM-CSF, Wnt pathway, and IL-18 or IL-33 [92–95]. Evidence from the DSS colitis model has led to interests in the development of anti-IL-18 therapy [96], which will be evaluated in clinical trials (NCT03681067). Furthermore, the interplay of host and microbiome in colitis has been studied in this model [97]. One interesting modification of the DSS colitis model is the administration of azoxymethane before inducing DSS colitis. In this model, mice develop premalignant and malignant colorectal lesions, serving as a mirror for UC-associated colorectal cancer [98].

### 2.2. Chemical Models: Haptenating Agents

In these models, topical administration of a chemical agent leads to a modification of intestinal proteins triggering an immune response. Therefore, adaptive immune response is involved, although it has been shown that innate mechanisms are essential in these models, too [99, 100].

In the TNBS colitis model, trinitrobenzene sulfonic acid (TNBS) is applied directly into the colon in a mixture with ethanol that allows the TNBS to cross the epithelial barrier. Mice then develop severe diarrhea, a wasting syndrome and rectal prolapse with maximum severity after about 3 weeks [99]. In this model, some CD-like features as transmural inflammation, fibrosis, and an IFN*γ*- and Th1-dominant inflammation can be found. In its first publication, the essential contribution of IL-12 was already shown, with important therapeutic implications [101]. Since this first description was based on targeting of p40 and therefore suppressing IL-12 and IL-23 action, the contribution of each of those cytokines is still not completely understood in human disease, but in TNBS colitis, suppressing IL-23 unleashes IL-12 production via IL-17A and T-bet and therefore exacerbates colitis; so in this model, Th1 action seems to dominate [102, 103]. Subsequently, IL-12 and IL-23 were found to be the key regulators of Th17 differentiation. In a bedside setting, despite strong experimental evidence, disruption of IL-17A signaling by anti-IL-17A antibody secukinumab led to acute exacerbations of CD [104, 105]. While *summa summarum*, this cytokine therefore seems to have a rather protective function; targeting the Th17 differentiation was far more successful. Th17 cells differentiate under the influence of IL-23, IL-1*β*, IL-6, and TGF-*β* [106]. Targeting p40, a subunit of IL-12 and IL-23, by ustekinumab showed significant effects in two RCT of MC patients and is approved for therapy [107]. Risankizumab, targeting specifically IL-23, showed promising results in earlier clinical trials [108, 109]. One more recently described cytokine, TL1a, is involved in TNBS colitis and associated fibrosis and has recently been found to be a beneficial target in IBD models, including treatment of fibrosis [110]. PF-06480605, a TL1a targeting agent, is currently under clinical phase II evaluation (NCT02840721). Adhesion molecules of the alpha-4-integrin pathway have been evaluated in the treatment of IBD, but unfortunately, unselective block of alpha-4-integrin by natalizumab led to reports of lethal viral encephalopathy. The *α*4*β*7-integrin-MAdCAM-1 pathway was subsequently identified as the gut-selective subtype of leucocyte adhesion, and interference showed a significant therapeutic effect in TNBS colitis [111], eventually leading to the development of vedolizumab, an *α*4*β*7-integrin antibody approved for IBD therapy. TNBS can be used to provoke a more acute colitis as described above or induce a chronic colitis, when repeatedly administered in low doses. Importantly, this chronic inflammation is also self-limited despite continued TNBS application and gives insights into the mechanisms of acquired immune tolerance [112–114].

Oxazolone is another such agent and is also administered topically. The colitis evoked by oxazolone is superficial, leading to edema and ulcers as well as neutrophil and lymphocyte infiltration; therefore, it has many similarities with UC [53, 115]. Both oxazolone colitis and UC are characterized by increased IL-9 and IL-13 production [116]; the latter has been shown to be essential in oxazolone colitis and is produced by the equally essential NKT cells. Differences between the model and the disease became apparent, however, when targeting IL-13 in UC failed to ameliorate disease activity. Anrukinzumab and tralokinumab, monoclonal antibodies against IL-13, as well as QAX576, an IL-13 inhibitor, reached the phase II and phase I level of clinical evaluation for UC and CD, and further evaluation was discontinued because of discouraging results [57, 59, 117].

### 2.3. Mono- and Oligogenetic Models

There is a number of genetically engineered mice that develop spontaneous inflammation of the gut and are used as models for IBD [118]. Because of the highly specific alteration in these mice, the functional involvement of the target gene can be explored quite exactly, but these mice are less likely to resemble the human IBD. Altering genes involved in epithelial cell homeostasis and barrier function, mucin components, or anti-inflammatory pathways lead to spontaneous inflammation of the gut [119–126]. For example, mice with IL-10 deficiency spontaneously develop colic inflammation. Notably, some genetic models with a terminal ileitis and even skip lesions have been developed, resembling the most common distribution of inflammation in CD [127–131].

### 2.4. Adoptive Transfer Colitis

When naïve lymphocytes from syngenic donors are transferred to a SCID- or *Rag1*^−/−^ mouse, a severe colitis with weight loss develops. This is due to a lack of Tregs in this setting; transfer of mature Treg-containing lymphocytes or a cotransfer with Tregs prevents colitis [132]. Therefore, this adoptive transfer model is tremendously helpful for deciphering the mechanisms by which Tregs suppress inflammation [133]. Basically, by introducing further genetic modifications into donor or recipient mice, involvement of these genes can be shown; for example, the involvement of IL-10 and TGF*β* in the regulatory T cell function could be shown: disruption of IL-10, its receptor IL-10R*β*, TGF*β*, or expression of a dominant negative TGF*β*-receptor led to a loss of the suppressive action of Treg transfer in this model [134–137]. Further knowledge about Treg induction and stability by IL-23 and IL-33 has been demonstrated in this model [138, 139]. Furthermore, the essential contributions of IFN*γ* and of the Th17 response for developing transfer colitis could be shown using this approach by jamming T-bet or IL-23 and IL-17, respectively [140–143]. Despite the strong experimental evidence for IFN*γ* as a key driver of inflammation, targeting IFN*γ* in the clinical setting was not successful, as a phase II trial of fontolizumab, an anti-IFN*γ*-antibody, failed to meet the primary endpoint [144], but this study showed significant efficacy after longer treatment. SMAD7, a negative regulator of the TGF*β* pathway overexpressed in IBD, was evaluated as a therapeutic target using the adoptive transfer colitis model [69], and mongersen, a SMAD7 antisense oligonucleotide, showed promising results in phase II trials; unfortunately, the phase III trial lacked emerging benefit, leading to its termination (NCT02596893). One further important finding in this model was the plasticity of the Th response. Th17 cells were shown to be able to transdifferentiate to Th1 cells. Given this plasticity, we start to understand why some patients are not responsive to therapies targeting those different pathways specifically [75, 145].

### 2.5. Congenic Models

Some animal models develop an IBD-like phenotype spontaneously without a targeted intervention; they can be developed through specific breeding and since have a polygenetic and complex pathogenesis but therefore are thought to resemble human disease to the closest. One good example of this type of model is the SAMP1/YitFc mouse strain [129]. The ancestors of these mice have been held in scientific hands since almost one century now, with dramatic changes in its phenotype from a leukemia prone to a premature senescent phenotype and now to the SAMP1/YitFc mouse. These mice spontaneously exhibit typical symptoms of CD-like skip lesions, ileitis, transmural and stricturing inflammation, perianal fistulizing disease, and extraintestinal symptoms [121, 129, 146, 147]. Since this phenotype developed through untargeted recombination, the responsible genomic loci were unknown and candidate loci were confirmed through generation of congenic strains, meaning that mice differing in only few genomic loci were bred and the impact of these differences was studied. This approach led to the identification of at least 4 highly IBD phenotype-associated loci, with strongest evidence for a locus spanning genes for IL-10 receptor alpha and IL-18, and this locus overlaps with susceptibility loci discovered in the DSS and TNBS colitis models [148–150]. One major advantage of this model is that subliminal changes preceding the onset of overt IBD can be studied in detail; in this model, alterations in epithelial composition and permeability could be observed prior to the onset of inflammation, suggestive of a causal role [129]. The SAMP1/YitFc strain paralleled the clinically observed response to anti-TNF*α* treatment and gave insights in the mechanism of action of these drugs in IBD [151].

## 3. Organoids

The models described above are suitable for reflecting the complex mechanisms in a whole organism in an IBD-like condition, but need mice to be raised for weeks and to be killed and lack many of the conveniences of cultured cells, which are much easier to manipulate, to observe, and to analyze; furthermore, the murine origin can impair the validity for human disease. Cell cultures, on the other hand, mostly stem from malignant transformed cells, are therefore unlikely to reflect the behavior of healthy cells, especially if one keeps in mind that one of the hallmarks of malignant tumors is to stop forming an intact epithelial monolayer, whereas the integrity of the latter is one of the key focusses in IBD research. Primary epithelial cells do not form stable cultures *in vitro*, making their use difficult.

The cornerstone for circumvention of these problems was laid recently with the development of intestinal organoids. Organoids can now be raised from stem cells, either by differentiation of pluripotent stem cells or by culture of adult intestinal stem cells from isolated intestinal crypts under specific conditions. In 2009, Sato et al. published a report of successful differentiation of single murine intestinal stem cells towards crypt-villus domain containing spherical bodies, using specific culture conditions. The group had discovered a stem cell specific marker, Lgr5, before. These Lgr5^+^ cells then could be grown in a 3D basement membrane-like gel called Matrigel, which contains the secretion of a sarcoma cell line and contains many of the basement membrane proteins. Combined stimulation with R-spondin 1, EGF, and Noggin-like peptide then led to the formation of self-organizing bubble-like epithelial structures with crypt-villus domains, stem cells in the bottom of the crypt, Paneth cells, enteroendocrine cells, and goblet cells. Villi protruded into the lumen of these organoids, which was filled with apoptotic cells shedding from the villus tips. These organoids were stable in culture, and a closely related technique could be used to establish indefinitely growing organoids from human colonic epithelium [152–154]. Published in 2011, Spence et al. were able to direct the differentiation of human-induced pluripotent stem cells and embryonic pluripotent stem cells towards intestinal organoids containing all intestinal epithelial cell types, crypts, microvilli, and transepithelial substance transporting properties [155]. In short, Activin A induced the formation of definitive endoderm- (DE-) like cells from pluripotent stem cells. After 3 days of treatment, the cells had acquired the DE phenotype and were still able to form foregut and hindgut lineages. At this point of time, exposition to FGF4 and Wnt3a for 4 days led to stable induction of a hindgut phenotype. Cells formed tubes and budded off to hindgut spheroids; this resembled the embryonic hindgut formation closely. Those still cuboid, but on a molecular level clearly hindgut differentiated epithelial spheroids then matured in a Matrigel-based 3D culture system containing additional growth factors; this led to the formation of organoids resembling mature intestinal epithelium. In this model, even mesenchyme was formed. This pioneering work has now equipped scientists with a new model of intestinal epithelium that combines human origin, nonmalignant genetics, and accessibility to in vitro cell culture techniques as well as inclusion of all epithelial cell types present in normal epithelium including rare cells like M, Tuft, or enteroendocrine cells [156–158]. The latter is of importance, since these cells have been implicated in IBD pathogenesis but are difficult to assess in other models [159–161]. Furthermore, and unlike embryonic stem cells, this model can be generated easily from affected individuals as well as healthy controls, since only a small mucosal biopsy is needed and therefore is of lesser ethical concern. While organoids from iPSCs are of great interest in other fields of research, organoids from intestinal adult stem cells carry the advantage of genetic and epigenetic stability relative to the site of origin, while differentiation of iPSCs is linked to genetic and epigenetic variation [159–163]. These organoids are a relatively new tool, and some challenges must be encountered in the future. Pure epithelial organoids will barely be suitable to investigate interactions with other cell types, so modelling fibrosis or immune cell and vascular or neuronal interactions need a more complex model. Accordingly, coculture with other cell types has been developed. Nozaki et al. were able to add intraepithelial lymphocytes (IELs) to murine intestinal organoids, maintain these in culture, and perform stimulation and motility experiments on them. Culture of IEL was not possible before, and therefore, intestinal organoids opened the window for detailed research on IELs [164, 165]. Pastuła et al. successfully added fibroblasts and enteric nerves to these models and therefore were able to modulate the intestinal stem cell niche more precisely, giving the opportunity for the investigation of the role of these interactions in carcinogenesis or wound healing [166]. A coculture system adding macrophages was developed by Noel et al. In this model, importantly, an increase in mucosal barrier function and interactions of epithelial cells and macrophages were observed, and a coordinated response to pathogenic strains of *E. coli* could be shown in vitro [167]. In this latter work, the bubble-like organoids were grown in polarized 2D cultures, as it had been developed before [168]. This is of great importance, since in the bubble-like structures access to the apical surface of the artificial epithelium is difficult, but as outlined above, the luminal contents of the bowel are thought to be indispensable for IBD pathogenesis. In these 2D cultures, however, both sides of the artificial epithelium are directly accessible; furthermore, the growth on a liquid-air interface has been shown to be important for gene expression. These 2D cultures were shown to develop abilities similar to the gut epithelium, like IgA transcytosis, peptide absorption, or polarized cytokine secretion [169]. Furthermore, interaction with apical bacteria and viruses has already been modulated [168, 170–172]. Adding even more complexity, microfluidic organ-on-a-chip models have been developed, adding vascular cells, bacterial flora, and even flow and mechanical forces like in human bowel to the model; these highly developed systems are very promising for the development of pharmacological high-throughput compound screening [173].

**Figure 1:**
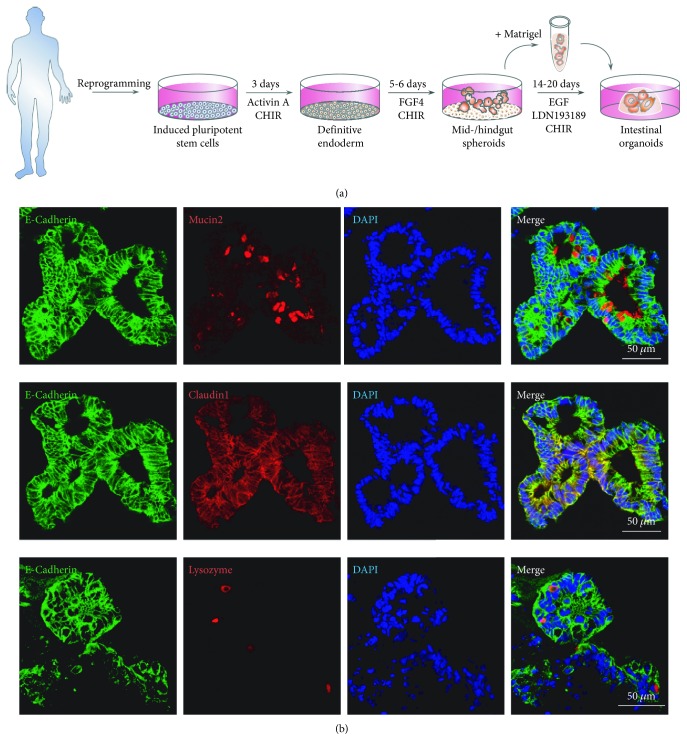
(a) Reprogramming of somatic cells towards induced pluripotent stem cells (iPSCs) and differentiation of iPSC into intestinal organoids using different culture conditions. (b) Immunofluorescence staining of induced human intestinal organoids for essential markers of intestinal differentiation. After evolution through the protocol shown in (a), organoids express mucin, claudin 1, and lysozyme together with E-cadherin.

Rare forms of IBD, especially those with very early onset in early childhood, are caused by monogenetic aberrations, for example, in IL-10, IL-10R, XIAP, NCF2, or TTC7 [174]. Growing organoids from these young patients can help understand the role of those genes, as it was recently the case with caspase 8 [175] or NOX1 defects [176], but did also lead to the discovery of compounds that were able to reverse the morbid consequences of those mutations. For example, intestinal organoids grown from children with multiple intestinal atresia, a rare congenital condition with IBD-like features, showed an inverted growth with the cells apical side on the outside of the organoids; treatment with a Rho kinase inhibitor led to reversal of this inversion and could help in the development of new therapies for this condition [177]. Besides those monogenetic conditions, more than 200 risk loci have been found associated with IBD. In addition to the frequently used genetically manipulated mice, intestinal organoids are increasingly used to elucidate the role of the IBD-associated genes. For example, the interplay of IL-22 and the risk gene ATG16L1 was recently discovered using intestinal organoids [178]. Hohwieler et al. have recently described the generation of induced human intestinal organoids (iHIOs) derived from a patient with a severe course of CD. These organoids were derived from keratinocytes via generation of induced pluripotent stem cells and subsequent stepwise directed differentiation towards intestinal organoids. No significant differences between patient derived and control cells from healthy donors could be observed within the course of directed differentiation from iPSCs towards iHIOs. Upon continued organoid culture, a decreased amount of goblet cells could be observed. This aspect may rise interest as defective goblet cell differentiation has previously been described for UC [179] (see Figure 1).

As described above, common IBDs are thought to be multifactorial diseases with a polygenetic predisposition; there is a lack of models with an authentic epigenetic background. Organoid cultures have been successfully established from the inflamed mucosa of IBD patients; subsequent analyses have proven that transcriptional signatures of inflamed epithelial structures may persist within descending organoids [180, 181]. Therefore, organoid-based models of IBD open further fields of research for scientists: epigenetic changes have recently been involved in IBD pathogenesis [182–186]. Howell et al. then showed in a very robust study that besides site-specific epigenetic imprintings, there are disease-specific epigenetic alterations in pediatric IBD epithelial cells at disease onset and these seem to persist even after the inflammation passed by. Importantly, many of the epigenetic changes observed in IBD correlate with known genetic susceptibility loci. Epigenetic alterations are difficult to analyze on a functional level, but importantly, the group was also able to show that organoids derived from these IBD patients retained their epigenetic characteristics. Therefore, intestinal organoids derived from affected individuals could be new promising models to investigate the effects of these epigenetic changes on a functional level and the influence of novel therapeutics on them [187]. Vice versa, since heterotopic transplant experiments in mice have shown phenotypical stability of intestinal organoids even after transplant in another bowel segment, healthy organoids could be a first step to a “mucosal transplant” for IBD [188–190].

Latest work from the Jonkers Lab illustrates additional options of organoid-based disease modelling in IBD research: patient-derived organoids displayed an effective epithelial barrier. Significant disruption of the epithelial barrier function could be induced by EGTA: diffusion of fluorescein isothiocyanate-labelled dextran of 4 kDa (FITC-D4) towards the intestinal organoids lumen was markedly increased in IBD-derived organoids upon EGTA exposure analog, the lines of Caco-2 cell monolayers. These findings suggest that intestinal organoids might have substantial value for disease modelling of disturbed epithelial barriers [181].

**Figure 2:**
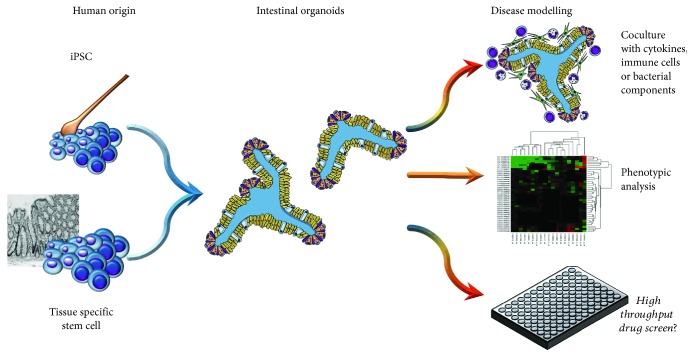
Derivation and potential use of intestinal organoids in IBD research. Induced pluripotent stem cells (iPSCs) can be derived from somatic cells, e.g., from pulled hair keratinocytes. Intestinal organoids then can be grown from iPSCs or from intestinal crypts and be used for disease modelling by coculture with inflammatory cells or bacteria, exposition to signaling molecules, or phenotypic analysis. Future applications could be the development of high-throughput drug screening assays.

Many important laboratory techniques have been used on organoids with success. Since freezing and thawing techniques also work for organoids, van de Wetering et al. started up a “living biobank” with cryopreserved organoids from colorectal cancers and associated normal epithelium. As the authors outline, this could be of high importance for drug development and ex vivo drug susceptibility testing for patients with CRC [191], and the same could be true for patients with IBD and other epithelial gut diseases. Given the stability of organoid cultures, even high-throughput compound screening seems possible (Figure 2). Genetic editing and gene silencing have also been performed successfully in organoid cultures [192–195], making this model even more versatile.

Extensive fibrosis is one of the major complications of CD. The mechanisms behind this process are deciphered only in part, and using in vitro organoid models could prove to be helpful. As described above, this mesenchymal reaction could be modelled by raising intestinal organoids from PSCs or by coculture with mesenchymal cells, but recently, epithelial to mesenchymal transition (EMT) was induced in intestinal organoids from mice, and TGF*β* and TNF*α* were shown to induce this EMT [196]; both cytokines have been implicated in IBD pathogenesis. Further development of this model could be helpful for understanding the process of fibrosis in an IBD background.

## 4. Conclusions

Inflammatory bowel diseases cause high morbidity and substantial costs. Our knowledge about the causes of those diseases is rapidly growing, leading to new therapeutic options, but since still not every IBD can be controlled today, further work is needed to understand the complex pathogenesis and find novel treatments. Much of our current knowledge comes from mouse models, genomic association studies, and rare monogenetic conditions with an IBD-like phenotype; in addition, molecular studies have been performed in vitro, mostly using transformed epithelial cell lines, because primary intestinal epithelial cells could not be grown in a stable culture. Progress in the exploration of the pathogenesis was hampered by some substantial disadvantages of the models, which had genetic differences due to malignant or murine origin, were time-consuming to create, difficult to manipulate, and hardly accessible for high-throughput or living tissue setups. The recent development of intestinal organoid cultures that can be easily derived from healthy or affected persons and manipulated with any laboratory in vitro method overcame those disadvantages and adds a highly promising model to the benches of scientists and has already led to new insights into the puzzle of IBD. Further development of organoid-based methods is likely to revolutionize the field of IBD research; challenges are the addition of further tissue components like vessels, nerves, and stroma and the stable coculture with an intestinal-like microbial flora.
